# Weight Regain and Insufficient Weight Loss After Bariatric Surgery: Definitions, Prevalence, Mechanisms, Predictors, Prevention and Management Strategies, and Knowledge Gaps—a Scoping Review

**DOI:** 10.1007/s11695-020-05160-5

**Published:** 2021-02-08

**Authors:** Walid El Ansari, Wahiba Elhag

**Affiliations:** 1 Department of Surgery, Hamad General Hospital, Doha, 3050 Qatar; 2 College of Medicine, Qatar University, Doha, Qatar; 3 Schools of Health and Education, University of Skovde, Skövde, Sweden; 4 Department of Bariatric Surgery/Bariatric Medicine, Hamad General Hospital, Doha, 3050 Qatar

**Keywords:** Weight regain, Insufficient weight loss, Bariatric surgery, Definitions, Mechanisms, Predictors, Prevention, Management

## Abstract

Some patients experience weight regain (WR) or insufficient weight loss (IWL) after bariatric surgery (BS). We undertook a scoping review of WR and IWL after BS. We searched electronic databases for studies addressing the definitions, prevalence, mechanisms, clinical significance, preoperative predictors, and preventive and treatment approaches including behavioral, pharmacological, and surgical management strategies of WR and IWL. Many definitions exist for WR, less so for IWL, resulting in inconsistencies in the reported prevalence of these two conditions. Mechanisms and preoperative predictors contributing to WR are complex and multifactorial. A range of the current knowledge gaps are identified and questions that need to be addressed are outlined. Therefore, there is an urgent need to address these knowledge gaps for a better evidence base that would guide patient counseling, selection, and lead to improved outcomes.

## Introduction

Bariatric surgery (BS) is currently the most efficacious and durable intervention for severe obesity. Unfortunately, some degree of weight regain (WR) is common after patients reach their nadir weight, where about 20–25% of patients struggle with considerable WR after BS [1–5]. Likewise, insufficient weight loss (IWL) (< 50% EWL) was the most common reason to qualify for revisional BS [6].

Some authors have proposed behavioral and biological mechanisms for WR [1], but the preoperative factors that predispose patients to significant WR remain unclear. Identifying these factors could improve the counseling of patients regarding the prevention of WR [2]. It is also important to mention that WR is associated with the deterioration of the quality of life and the reappearance or worsening of obesity-associated comorbidities, e.g., hypertension and type 2 diabetes (T2D) which necessitate close monitoring and appropriate management [7, 8]. Moreover, revisional BS to manage WR/IWL may have higher complication and mortality rates compared to primary BS [9–12].

The literature reveals gaps. Previous reviews focused mainly on the prevention strategies that improve weight loss [13, 14]. However, in terms of management approaches, one meta-analysis assessed surgical revision for the management of WR following BS [3]. Another scoping review explored various treatment options including behavioral, psychotherapeutic, and pharmacological interventions [15]. To the best of our knowledge, no previous study examined the broader landscape of WR and IWL following BS, to include the definitions, prevalence, causes, predictors, implications, prevention approaches, and management strategies.

Therefore, in order to address these knowledge gaps, the current scoping review explored the definitions, prevalence, causes, preoperative predictors, implications, prevention and management strategies of WR and IWL following BS. Given the obesity epidemic and frequency of BS as a treatment strategy, WR/IWL after BS requires a clear understanding in order to prevent patient dissatisfaction, lower quality of life, and relapse of comorbidities. These considerations inspired the current scoping review.

## Methods

The goal of a scoping review is not to locate, recover, and include every single published report on the subject. Rather, the aim is deliberately broader in order to explore the literature, ascertain the essential characteristics of the subject, discover the likely gaps, and demonstrate critical examples. A scoping review can establish the types of prevailing evidence in a given field, the critical features related to a specified topic, or the underlying knowledge gaps. Scoping reviews are beneficial for broad questions, e.g., “What information exist on this topic?” and for assembling and evaluating information before commencing a systematic review. A scoping review is advantageous when the information on a topic has not been comprehensively reviewed or is complicated and diverse, as the broad extent of the information renders applying formal meta-analytic processes unachievable.

Thus, the scoping review was chosen to appraise WR after BS, a compound and diverse topic that demands extensive coverage. We utilized a six-step framework for scoping reviews (emphasized below) and applied its criteria that included identifying the research question/s; identifying relevant studies; study selection; charting the data (data extraction process); collating, summarizing, and reporting the results; and (optional step 6) a consultation exercise.

### Research Questions

The present review “scoped” the literature in order to respond to six questions related to WR and IWL following BS: (1) What are the definition/s?; (2) What is the reported prevalence?; (3) What are the possible mechanisms and preoperative predictors?; (4) What are the clinical impacts and implications?; (5) What are the prevention and management strategies?; and, given the answers to the first 5 questions, (6) what are the knowledge gaps and possible ways forward?

### Identifying Relevant Studies

*Information sources*: We searched electronic databases including PubMed, MEDLINE, Embase, CINAHL, Web of Science, and Scopus, as well as Google scholar for published articles of all types of WR/IWL and their relationships with any type of BS. *Keywords and search terms*: keywords were “bariatric surgery” [in Title/Abstract]. The medical subject heading (MeSH) terms used were bariatric surgery [All Fields] AND (“weight regain” [MeSH Terms]; bariatric surgery [All Fields] AND (“weight gain” [MeSH Terms]; bariatric surgery [All Fields] AND (“insufficient weight loss” [MeSH Terms]; bariatric surgery [All Fields] AND (“postoperative AND insufficient weight loss”[MeSH Terms]; bariatric surgery [All Fields] AND (“postoperative AND weight regain” [MeSH Terms]; bariatric surgery [All Fields] AND (“postoperative AND weight gain” [MeSH Terms]. As the retrieved literature uncovered more features about post-BS WR/IWL, supplementary searches were formulated and conducted to obtain the literature related to the uncovered features.

### Study Selection

Inclusion criteria included original studies; published in English; from 1 January 1994 through 30 September 2020; that assessed BS, WR/IWL; and enrolled patients of any age, gender, and ethnicity. Exclusion criteria comprised studies that did not include BS or WR/IWL.

### Charting the Data

The data extracted consisted of items relevant to the five research questions being examined.

### Collating, Summarizing, and Reporting the Results

The review team gathered, organized, and summarized the findings and report it below. Based on the emergent findings, we mapped the potential gaps that, if addressed, could present opportunities for advancing the field.

### Consultation Exercise

Two experts (senior consultants, bariatric surgery and bariatric medicine) reviewed the findings to advise on and corroborate the findings of the current review.

#### Definition/s of WR and IWL after BS

In order to answer the first research question, there is a need to have a clear differentiation between two types of weight loss failure: IWL and WR. Insufficient weight loss is defined as excess weight loss percentage (EWL%) of < 50% 18 months post-BS [16]. WR is defined as progressive weight regain that occurs after achievement of an initial successful weight loss (defined as EWL>50%) [16]. The existing inconsistency, multiplicity, and lack of a standardized definition of WR (Table 1) lead to poor reporting and understanding of the clinical significance of WR [8, 16, 37, 38]. For example, the use of 5 continuous and 8 dichotomous measures among 1406 patients reported WR rates that ranged between 44 and 87% five years post Roux-en-Y gastric bypass (RYGB) [8]. Moreover, the percentage of maximum weight lost had the strongest association with the progression of T2D and hypertension, reduced quality of life, and reduced satisfaction with surgery [8]. Others observed a 16–37% WR five years after BS, where the three WR definitions examined were found to be associated with deterioration in quality of life; however, no associations were found with other comorbidities [23]. Therefore, more research is required to assess the extent of clinically significant WR that would indicate when an intervention is required and guide the type of intervention [16]. As for IWL, despite its distinct definition, it has been much less assessed, being only informally referred to in association with WR [16].

**Table 1:** Selected examples of definitions and prevalence of WR and IWL after BS

Characteristic	Unit/component/s	Examples
Definition		
WR	Using EWL%	> 25% EWL from nadir [17–19]
	Using nadir weight %	≥ 10% [8, 20] or > 15% of nadir weight [8, 9, 21, 22]
	Using nadir weight kg	≥ 10 kg from nadir [8, 21–23]
	Using maximum WL	≥ 10% [8, 24], ≥ 20 [8, 25] or ≥ 25 [8, 26] of maximum WL
	Using pre-surgery weight	≥ 10% WR of pre-surgery weight [8, 27]
	Using any WR after remission	Any WR after T2DM remission [28]
	Using any WR	Any WR [29]
	Using BMI	≥ 5 BMI kg/m^2^ points from nadir [30]
		Increase in BMI > 35 kg/m^2^ after successful WL [31]
IWL	Using EWL%	EWL of < 50% at 18 months [16]
Prevalence^a^		
WR		Post-LAGB (38%) [32]; post-LSG (27.8%) [33]; post-RYGB (3.9%) [34]
IWL		After LSG (32–40%) [17, 35]; after RYGB, OAGB, and LSG combined (20%) [36]

Range of definitions and prevalence selected are examples for illustration purposes only and do not include all examples in the literature. *EWL* excess weight loss, *WR* weight regain, *IWL* insufficient weight loss, *WL* weight loss, *T2DM* type 2 diabetes, *BMI* body mass index, *LAGB* laparoscopic adjustable gastric banding, *LSG* laparoscopic sleeve gastrectomy, *OAGB* one anastomosis gastric bypass

^a^Prevalence of WR are different depending on choice of BS procedure, varied assessment methods (EWL, weight from Nadir), and various follow-up periods

#### What Is the Reported Prevalence of WR and IWL After BS?

In terms of the second research question, WR following BS varies by the type of BS performed, whether restrictive and/or malabsorptive. Table 1 shows the variations in the reported prevalence of the two outcomes that the current review examined. A large prospective multicenter Swedish study found that 10 years after laparoscopic adjustable gastric banding (LAGB), patients regained 38% of the maximal weight they lost at 1 year [32], and WR after laparoscopic sleeve gastrectomy (LSG) was 27.8% (range 14–37%) at long-term follow-up (≥ 7 years) [33]. On the other hand, the Longitudinal Assessment of Bariatric Surgery (LABS) study reported a 3.9% WR 3–7 years after RYGB [34].

In comparison with WR, data on the prevalence of IWL is more limited [36], mostly assessed or stated as a “spin-off” when discussing the indication for revisional surgeries [17, 39]. For instance, among 17 patients who underwent revision after LSG, 40% were indicated for conversion to biliopancreatic diversion/duodenal switch (BPD/DS) and RYGB because of IWL [17], and others found that 32% of patients underwent revisional RYGB because of IWL [35].

#### Mechanisms of WR

As regards the third research question, several mechanisms contribute to WR following BS (Table 2). These include hormonal mechanisms, nutritional non-adherence, physical inactivity, mental health causes, and maladaptive eating [3, 37]. Surgical mechanisms are also implicated in WR, e.g., enlargement of gastric pouch, stoma dilatation, or gastrogastric fistula. Table 2 provides a summary of the mechanisms of WR and IWL.

**Table 2:** Summary of causes, predictors, and prevention and management strategies of WR and IWL after BS

Characteristic	Summary
Causes	
Hormonal/metabolic	Increase in ghrelin, decrease in peptide YY and GLP-1, post-bariatric hypoglycemia, role of leptin is unclear [24, 40–49]
Dietary non-adherence	Increase caloric intake with time, dietary non-adherence/food indiscretion, grazing, lack of nutritional follow-up [13, 32, 50–56]
Physical inactivity	Non-compliance, sedentary behavior, presence of barriers to exercise [51, 57–61]
Mental health	Depression, multiple psychiatric conditions, binge eating disorder, loss of control over eating [54, 62–68]
Anatomic surgical failure	
LAGB	Pouch distension [69]
LSG	Dilatation of gastric pouch [70–77]
RYGB	Dilatation of gastric pouch, dilatation of gastrojejunostomy stoma outlet, gastrogastric fistula [73–75]
Predictors	Older age, male gender, higher preoperative BMI, mental health issues, presence of comorbidities (T2DM, hypertension, OSA) [34, 36, 76–86]
Prevention and management	
Behavioral	Cognitive behavioral therapy, remote acceptance-based behavioral intervention, lifestyle counseling [87–90]
Dietary	Counseling with dietitian, structured dietary intervention [91–94]
Pharmacological	FDA approved: phentermine, phentermine–topiramate extended release, liraglutide, bupropion/naltrexone
	Off label: metformin, topiramate, zonisamide, bupropion [95–98]
Surgical (management only)	
After failed LAGB	Conversion to LSG, RYGB, BPD/DS [99]
After failed LSG	Conversion to RYGB, BPD/DS [17]
After failed RYGB	Conversion to DRYGB or to BPD/DS; or revision of gastric pouch and anastomosis, revision with gastric band [100]

*WR* weight regain, *IWL* insufficient weight loss, *BS* bariatric surgery, *GLP-1* glucagon-like protein-1, *LAGB* laparoscopic adjustable gastric banding, *LSG* laparoscopic sleeve gastrectomy, *RYGB* Roux-en-Y gastric bypass, *BPD/DS* biliopancreatic diversion with duodenal switch, *FDA* Food and Drug Administration, *DRYGB* distal RYGB

##### Hormonal and Metabolic

Weight loss after BS is due to the anatomical exclusion of the foregut. This leads to a hormonal upregulation of pancreatic peptide YY, glucagon-like peptide-1 (GLP-1), and gastric inhibitory polypeptide hormones which promote satiety and minimize hunger, as well as downregulation of ghrelin with subsequent decrease in food intake [40, 41]. With time, alterations in the levels of ghrelin, leptin, and incretins diminish, resulting in WR [42–44].

For ghrelin, one study found that RYGB patients with WR had significantly higher ghrelin before and 2 years after surgery compared to those who maintained or lost weight (722 ± 29 vs 540 ± 156 pg/ml) [45]. Similar findings were observed among patients with WR 5 years after LSG [42]. Likewise, after RYGB, gastric inhibitory polypeptide and GLP-1 were lower among WR patients compared with patients who successfully maintained WL [43]. As for PYY, rodent studies showed that postsurgical WR was associated with failure to maintain elevated plasma PYY concentrations [46]. However, the role of leptin in WR is not completely understood, with suggestions that the weight plateau after BS could be due to a decline in leptin level, despite that the administration of leptin did not result in significant weight reduction among women with WR after RYGB [47, 48]. Nevertheless, leptin administration may diminish sweet cravings [48].

Post-bariatric hypoglycemia is also associated with WR. This is a reactive hypoglycemia that occurs after carbohydrate intake, caused by the change in intestinal anatomy that leads to an exaggerated insulin surge. Glucose fluctuation causes hunger a few hours after a meal leading to frequent snacking. After RYGB, 54% of patients with WR experienced blood glucose levels consistent with hypoglycemia [24]. After LSG and RYGB, 79.2% of the patients exhibited 10.8% WR at 40 months following surgery, and the odds of ≥ 10% WR was found to be significantly more among those with post-BS hypoglycemia (OR = 1.66) [49]. Collectively such findings highlight the need for more research to explore the long-term effects of BS on the metabolic and gut hormonal regulation of WR.

##### Dietary Non-adherence

BS reduces the caloric intake in the immediate postoperative period due to the reduced gastric capacity, decreased hunger, and increased satiety. Nevertheless, for some patients, the caloric intake gradually increases, hence contributing to WR. In a Swedish study, the mean daily caloric intakes increased from1500 kcal/day at 6 months to 2000 kcal/day at 4–10 years post-BS, contributing to long-term WR [32]. Among RYGB patients, 23% demonstrated dietary non-adherence and continuation of their pre-surgical eating patterns, leading to suboptimal weight loss, WR, or both [50]. After BS, food indiscretion such as intake of excessive calories, snacks, sweets, oils, and fatty foods was statistically higher among patients with WR [51]. As for IWL, after RYGB, more than 50% of patients had IWL due to excessive consumption of high-calorie liquids [52]. Moreover, the incidence of intake of high-calorie liquids was higher among LAGB patients than among RYGB/LSG patients [52], probably because the intake of such liquids is more tolerated post-LAGB than after RYGB/LSG due to the anatomical restriction and dumping syndrome associated with each of these two procedures [13].

Grazing is defined as repeated episodes of consumption of smaller quantities of food over a long period of time, accompanied with feelings of loss of control [53, 54]. Due to the restrictive effect of BS, grazing is physiologically more possible than large binges. Grazing has been shown to be associated with poor weight loss outcomes post-BS. A meta-analysis of 994 post-BS subjects, mostly women, found that the prevalence of grazing was 16.6–46.6%, while the prevalence of WR was 47% [55]. Likewise, the lack of post-BS nutritional follow-up was significantly associated with WR [51], where 60% of patients with WR never maintained nutritional follow-up [56]. This highlights the importance of appropriate nutritional counseling after BS in order to prevent WR, and to ensure a long-term weight maintenance.

##### Physical Inactivity

Physical activity (PA) is associated with weight loss after BS [57, 58]. Although many patients increase their PA, most remain insufficiently active. Studies have shown that only 10–24% of post-BS patients met the PA guidelines for health promotion (i.e., ≥ 150 min/week or moderate-to-vigorous PA in bouts of ≥ 10 min) [59]. Moreover, the method used to measure PA could lead to conflicting results. For instance, a prospective study found that although PA significantly increased post-surgery based on participants’ subjective self-reported exercise diary, objective measurement of the PA showed that 24–29% of participants were less active postoperatively than preoperatively [60]. Inadequate PA and sedentary lifestyle also contributed to WR post-RYGB, where the incidence of WR was higher among patients who remained relatively inactive compared with patients who performed PA [51]. Addressing the barriers to exercise, e.g., health concerns, pain, lack of proximity to a gym or park, and feeling self-conscious, might have a positive effect on post-BS PA [60, 61].

##### Mental Health Conditions

Psychological factors might undermine WL by impeding motivation or hindering the compliance with diet, PA, and other behaviors that are critical to maintain WL [62]. For example, a study found that one year after RYGB/LAGB, psychiatric disease was associated with IWL (47.5%) and WR (29.5%) [63]. Moreover, patients with ≥ 2 psychiatric conditions were ≈ 6 times more likely to lose no further weight or exhibit WR relative to those with ≤ 1 psychiatric diagnosis [63]. Likewise, depressive disorders are associated with poorer WL, but the directionality remains uncertain [64]. For instance, depression was identified in 45%, 12%, and 13% of patients prior to RYGB, and at 6 and 12 months following surgery, and while preoperative depression did not predict postoperative weight outcomes, depressive symptoms after surgery predicted poor weight loss outcomes [65]. Similarly, 50% of patients had depressive symptoms at 22–132 months post-BS [65]. These symptoms were significantly related to WR, loss of control over eating (LOC), and concerns with body image [66]. Equally, binge eating may persist or remerge after BS despite the physical limitations of BS on the capacity of the stomach [54, 67]. Researchers found that binge eaters had a 5.3 kg/m^2^ increase in BMI compared to a 2.4 kg/m^2^ among non-binge eaters 2–7 years after RYGB [68]. In addition, LOC predicted poorer WL, where, at 12 and 24 months after RYGB, patients with LOC lost significantly less weight compared to those with no LOC [65].

##### Anatomic Surgical Failure

The mechanism by which surgical failure leads to WR varies by the type of BS. After LAGB, distension of the pouch hinders WL [69]. After LSG, dilation of gastric pouch is correlated with postoperative BMI [70, 71], where dilatation leads to the loss of restriction resulting in reduced satiety, increased food intake, and subsequent WR. Research has shown that the mean gastric volume in patients with WR increased from 120 ml early post-surgery to 524 ml at 5 years [72]. After RYGB, the dilatation of the gastric pouch or gastrojejunostomy (GJ) stoma outlet was associated with increased food intake and WR [73, 74]. Among RYGB patients who underwent upper endoscopy as workup for WR, researchers identified the dilation of the GJ in 58.9%, enlarged gastric pouch in 28.8%, and both abnormalities in 12.3% of the patients [73]. Stoma diameter (> 2 cm) was also independently associated with WR [74]. Gastrogastric fistula may also diminish the restrictive and malabsorptive components of RYGB leading to WR [75].

#### Preoperative Predictors of WR and IWL Post-BS

In terms of the fourth research question, very few studies have addressed the preoperative predictors of IWL after BS. Table 2 provides a summary of the predictors of post-BS WR and IWL.

For instance, higher preoperative BMI was associated with WR and a worse weight trajectory, where patients with baseline BMI ≥ 50 kg/m^2^ were more likely to have significant WR, while those with BMI < 50 kg/m^2^ were more likely to continue losing weight at 12 months post-surgery [76]. Similarly, among LSG patients with BMI > 40 kg/m^2^ prior to surgery, 80–100% of them reported WR two years after surgery [77]. Greater preoperative BMI was also significantly associated with suboptimal WL [78, 79].

Age seems to be another preoperative predictor of WR and IWL, but findings are inconsistent. Some studies found that older age (> 60 years) was associated with WR [80, 81]. Conversely, other research observed younger age to be more likely associated with WR [82]. As for IWL, older age predicted IWL one year after BS [36]. Likewise, research suggested that male gender was associated with suboptimal or worse WL after RYGB [34, 78].

Mental health conditions represent another preoperative predictor of WR and IWL. The diagnosis of binge eating disorder prior to surgery predicted higher BMI at 5 years [83]. In agreement, the lack of control of food urges and low self-reported well-being scores independently predicted WR at 28 months post-RYGB [84]. Interestingly, preoperative psychiatric disorder was a weaker predictor of WR than the postoperative psychiatric disorder, likely due to the impact of the latter on eating behavior after BS [85]. The association of depression and anxiety with IWL has been less examined; however, recent research found that both conditions significantly predicted IWL 1 year after BS [36].

The presence of comorbidities predicts WR and IWL. For instance, T2D predicted WR and IWL [34, 79–81, 86]; while hypertension and low HDL cholesterol were both associated with poor weight trajectory [34]. Similarly, the number of comorbidities and previous history of hypertension predicted IWL [36]. Likewise, patients with obstructive sleep apnea achieved significantly lower EWL% at 1 year after surgery than patients with no sleep apnea [86].

#### Prevention and Management Strategies of WR and IWL After BS

Due to the complex etiology of WR and IWL, a multidisciplinary approach to treatment ensures effective long-term success. Table 2 provides a summary of the prevention and management strategies of WR and IWL.

The management starts with a comprehensive assessment that includes dietary patterns, PA level, psychological disorders, and motivation [3, 15]. Education sessions provide patients with practical knowledge, skills, and support in order to make the necessary changes required for weight optimization. Dietary and PA counseling by a team of health professionals ensures that patients receive specialist-tailored advice. Mental health issues such as mood, anxiety, addiction, and personality disorders should be addressed and managed effectively [3, 14]. Upper gastrointestinal contrast studies and esophagogastroduodenoscopy (EGD) should be undertaken, where appropriate, to evaluate the gastrointestinal tract and provide essential data about the gastric remnant, gastrojejunal anastomosis size, presence of gastrogastric fistula, as well as the location and integrity of the gastric band [101].

The prevention and management strategies are essentially similar for WR and IWL, with the exception that prevention does not include a surgical component. The management options of WR include behavior interventions, pharmacotherapy, endoscopic interventions, and surgical revision.

##### Behavioral Therapy

The aim of behavioral therapy is to assist patients in making long-term changes through monitoring and modification of their eating and PA behaviors. This can be achieved by controlling the environmental cues and stimuli that trigger eating or sedentary behaviors.

*Management*: A 10-week behavioral intervention emphasizing psychological skills to stop WR was feasible, acceptable, with high retention and satisfaction rates among completers [87]. More importantly, WR was stopped and even reversed with this intervention [87]. Similarly, among WR patients post-RYGB, cognitive and dialectical behavior therapies for 6 weeks resulted in a significant decrease in weight (mean 1.6 ± 2.38 kg), as well as improvements in the depressive symptoms, grazing patterns, and binge eating episodes [88]. Likewise, an online and phone behavioral intervention showed feasibility, acceptability, efficacy, and high satisfaction (70% retention) [89]. Moreover, WR was reversed, as participants achieved a significant 5.1% WL that was maintained at 3 months [89]. Given such promising results, more research is required to assess the effectiveness of behavioral therapy for IWL after BS as there are almost no studies on the subject.

*Prevention*: A systemic review of 15 behavioral management studies after RYGB/LAGB (8 studies provided cognitive behavioral therapy, 7 studies provided group support) found that patients achieved greater WL than controls across both interventions [90].

##### Dietary Therapy

*Management*: Very few studies have addressed the dietary management of IWL after BS. As for dietary management of WR, a randomized controlled trial (RCT) among 144 post-RYGB patients found that a nutritional intervention that comprised education sessions with a dietitian every other week for 6 weeks resulted in significantly greater EWL% (80% vs 64%) and BMI reduction (6.48 ± 4.37 vs 3.63 ± 3.41) at 12 months compared with usual care [91]. Likewise, another RCT one year post RYGB used a structured dietary intervention that incorporated portion-controlled foods compared to usual care with both groups receiving behavioral WL instructions [92]. This RCT found that the intervention group had significantly reduced calorie intake at 4 months (− 108 vs − 116) and increased WL at 4 and 6 months (− 4.56% vs − 0.13%, − 4.07% vs − 0.14%, respectively) compared with the usual care group [92]. Similarly, a 16-week RCT among women who regained ≥ 5% of their lowest post-RYGB weight found that whey protein supplementation promoted WL (− 1.86 kg) and fat mass loss, with preservation of the muscle mass, compared to controls who gained weight (0.42 kg) [93]. Protein intake after BS preserves lean muscle mass, increases satiety, and improves and promotes WL [93].

*Prevention*: A study randomized 84 patients to dietary counseling (15 min, every other week, in person dietary counseling by a dietitian for the first 4 postoperative months) versus standard care (no formal nutrition counseling sessions) [94]. The dietary counseling patients lost more total WL percentage (TWL%) than those who received the standard care (20.7 ± 1.1% vs 18.5 ± 1.1%) [94].

##### Pharmacotherapy

In terms of management, several anti-obesity medications have been utilized in conjunction with lifestyle modifications in order to decrease hunger, promote satiety, and halt the WR after BS (Table 2). Research found that, among 319 patients with WR or inadequate WL post-RYGB or LSG, 54%, 30.3%, and 15% of the sample lost ≥ 5%, ≥ 10%, and ≥ 15% of their total body weight (TBW) respectively using medications [95]. Furthermore, patients who received topiramate were 1.9 times more likely to lose ≥ 10% of their TBW% compared to those who received other medications [95]. In the same study, regardless of the postoperative BMI, RYGB patients were significantly more likely to lose ≥ 5% of their weight with medications [95].

Other research that examined the use of topiramate, phentermine, and/or metformin among young adults with WR found that 54.1%, 34.3%, and 22.9% of the sample lost ≥ 5%, ≥ 10%, and ≥ 15% of their postsurgical weight, respectively, and those who used metformin demonstrated the highest percent weight change compared to the other medications [96]. This study also observed that patients who underwent RYGB achieved significantly higher TWL% compared to those who underwent LSG (− 8.1% vs − 3.3%) [96].

Likewise, a study of phentermine vs phentermine–topiramate extended release among post-RYGB/LAGB patients with WR and weight plateau found that phentermine and phentermine–topiramate patients lost 6.35 kg (12.8% EWL%) and 3.81 kg (12.9% EWL%) respectively, at 90 days [97]. This study also observed that patients receiving phentermine weighed significantly less than those on phentermine–topiramate throughout the study, with no reported serious side effects for both medications [97].

An evaluation of liraglutide (a GLP-1 analog that promotes WL) among 117 patients who undertook RYGB, LAGB, or LSG observed that patients achieved statistically significant WL (− 6.3 ± 7.7 kg) 7 months after receiving the drug, regardless of the type of surgery [98]. Moreover, the decrease in weight remained significant after 1 year of liraglutide 3 mg, and nausea was the most prevalent side effect (29.1% of patients) [98].

Generally, research on the use of prescription weight loss medications to treat WR or IWL is scarce and is primarily retrospective, and no studies were powered to determine the best medication/s or timing of introduction of the medication, during either the weight loss plateau or initial WR [102].

##### Surgical Revision

Revision of BS is indicated to address surgical complications, WR or IWL [103]. For instance, IWL was the cause of 68.03% of the conversion to LSG or RYGB after LAGB [99]. Both conversions were associated with comparable WL at 6 and 12 months, although %EWL and BMI reduction after 24 months were greater among the patients who underwent RYGB [99]. Similarly, others found that IWL and WR were the indications for revision to BPD/DS or RYGB after LSG [17]. The EWL% was significantly greater at 34 months for BPD/DS compared to RYGB (59% vs 23%), however the short-term complications and vitamin deficiencies were higher among the patients who underwent revisional BPD/DS [17]. A systematic review (799 studies) assessed the revisions of RYGB for WR, including two conversions (to distal RYGB or BPD/DS) and three revisions (revision of gastric pouch and anastomosis, revision with gastric band or with endoluminal procedures) [100]. The review found that the mean percentage excess body mass index loss (%EBMIL) at 3 years for these five revisional procedures was 52.2%, 76%, 14%, 47.3%, and 32.1%, respectively [100].

#### Now What? Knowledge Gaps and Possible Way Forward

Table 3 depicts the current knowledge gaps. Although WR/IWL is encountered after BS, their definitions are far from being standardized. Equally, our current understanding of the associations between the different definitions of WR/IWL and their subsequent clinical impact is limited, and practically, no data exists on the clinical significance of IWL. In addition, the extent and significance of the variety of mechanisms implicated in WR/IWL still require more certainty, and within this domain, the association of such mechanisms with IWL seems completely deficient. Likewise, the prevailing knowledge about the range of preoperative predictors of WR could be strengthened. Moreover, RCTs are needed to assess the current prevention and management strategies of WR/IWL. Table 4 outlines a possible way forward in terms of a range of research questions that need to be urgently addressed in order to progress the evidence base.

**Table 3:** IWL and WR after BS: area and extent of current knowledge gaps

Knowledge gap	Extent of gap^a^		Summary of potential gap
	WR	IWL	
Inconsistent reporting	+ +	+ + + +	Small sample sizes, patient recall to estimate nadir weight, loss to follow-up, and variability of follow-up times [8, 23]
Lack of standardization	+ + +	NA	Varied definitions, consensus statements, and guidelines of WR [8, 23]
Clinical significance	+ +		Relationships between different WR definitions and clinical outcomes require to be established [8, 23]
		+ + + +	No data on clinical significance of IWL, urgently needed
Limited data on			
Prevalence	+		Prevalence data mostly on WR [32–34]
		+ + +	Sparse data on prevalence of IWL [36], mostly assessed when discussing indication for revisional surgery [17, 35, 39]
Mechanism/s	+ + +		Small studies on WR
		+ + + +	Very sparse data on mechanism/s of IWL
Gut hormones	+ + +		Ghrelin, GLP, GIP: sparse data, small sample sizes; no long-term evidence [43]; PYY: only rodent studies [46]; leptin evaluated only in women [47, 48].
		+ + + +	Very few studies on gut hormones, leptin or PBH in relation to IWL
Dietary non-adherence	+ + +		Few small-sized prospective studies, more RCTs required [50, 51]
		+ + + +	Virtually no prospective studies on associations of caloric intake, macronutrient composition, dietary non-adherence, and food indiscretion with IWL
Physical in/activity	+ + +		Difficult to assess due to discrepancy between self-reported and measured PA [60, 61]; limited data on PA types, durations and levels and their associations with WR
		+ + + +	Very sparse data on PA types, durations and levels, and their associations with IWL
Mental health	+ + +		Relationship between preoperative depression and WR is unclear; research is required to establish the direction of the relationship [64, 66]
		+ + + +	Few reports on number of psychiatric diseases and loss of control over eating in relation to IWL [63, 65]; virtually no data on associations of depression and binge eating with IWL [68]
Surgical	+		Most studies on WR [72–75]
		+ + + +	Role of surgical causes in IWL practically not assessed
Management			
Behavioral	+ + +		Small studies with short follow-up in WR, no RTCs [87, 88]
		+ + + +	No prospective studies of patients with IWL
Dietary	+ + +		WR: few studies with small sample sizes and short durations (education sessions, structured dietary intervention); long-term, larger RCTs are needed [91, 92]
		+ + + +	No published data available on effects of dietary management in IWL
Pharmacological	+ + +		Small-sized retrospective observational studies, short follow-ups [95, 96, 98]; no robust RCTs to provide level 1 evidence of role of pharmacological approaches to WR [102]
		+ + + +	Effects of pharmacological therapy for IWL usually assessed in combination with WR [98, 102]
Surgical revision	+		Effects of surgical revision on weight usually assess WR and IWL combined [17]; no RCTs of the effects of various revisional surgeries on WR
		+ + + +	No RCTs of the effects of various revisional surgeries on IWL (for failed LAGB, LSG, RYGB) [99]

*WL* weight loss, *WR* weight regain, *IWL* insufficient weight loss, *RCT* randomized controlled trials, *PYY* peptide YY, *NA* not applicable, *PBH* post-bariatric hypoglycemia, *PA* physical activity

^a^The number of (+) signs signifies the extent of the current knowledge gap, where (+) suggests a small gap, while (+ + + +) indicates a large gap in the current available knowledge

**Table 4:** IWL and WR after BS: Research questions to enhance the evidence base

Topic area	Example
Defining the concept	Unit/s: What unit/s should be used to define WR/IWL? (e.g., nadir weight? EWL%?, kg?)
	Cutoff: Is there controlled (or acceptable) WR/IWL (e.g., 20–50 %WR from nadir after 2 years) and significant (or non-acceptable) WR/IWL?
	Definition: What is an appropriate definition of significant WR/IWL post-BS?
	Components: Should the appropriate definition be based solely on WR per se, or should it also incorporate element/s of the clinical implication/s resulting from WR/IWL? (e.g., recurrence of T2DM, HTN, dyslipidemia, deteriorated QoL?)
Prevalence	Based on the above, what is a “true” prevalence of WR/IWL after different types of BS?
	Separation: As WR and IWL have distinct definitions, should they be reported collectively or separately in future studies?
	Mandatory reporting: Should WR/IWL be one of the standard WL outcomes in comparisons of short-, medium-, and long-term outcomes of different types of BS?
Clinical outcomes	Generalization: Do WR/IWL always lead to recurrence of comorbidities? (e.g., why not all patients with WR experience recurrence of T2DM?)
	Extent: What are the impact/s of WR/IWL on changes in the status of different comorbidities?
	Patient/s: Is the extent of such impact/s different among patients (e.g., individualized to each patient)?
	Comorbidity/ities: Does the recurrence of a particular comorbidity/ities (e.g., T2DM, HTN, dyslipidemia, OSA) represent a sensitive “indicator” of the impact of WR/IWL?
Predictors	Known: Does addressing known pre-op predictors prevent WR/IWL or change their clinical outcomes? (e.g., lower BMI, younger age, earlier surgery)?
	Unknown: Are there additional modifiable/non-modifiable pre-op predictors of WR/IWL than already known (e.g., ghrelin, leptin)?
	Selection: Should such predictors guide the selection of the type of BS (e.g., malabsorptive surgery for higher BMI or patients with comorbidities such as T2DM)?
Mechanisms	Hormones: What is the precise role/s of various hormones (GLP-1, PPY, leptin) in WR and in IWL?
	Mental Health: What are the effect/s of maladaptive eating on WR/IWL (e.g., grazing, binge eating)?
	Psychiatric conditions: How do pre- and postop psychiatric illness affect WR/IWL (relationship/e.g., direction of depression and WR)?
	Physical activity: What is the precise role of PA in WR/IWL? How can PA be accurately assessed (e.g., discrepancy between objectively/subjectively measured PA)
	Surgery: How can the primary surgical technique be improved to prevent WR/IWL (e.g., biliopancreatic limb length)?
	Others: Are there other mechanisms that contribute to WR/IWL (e.g., exact role/s of gut microbiomes, bile acids)?
Management	
Behavioral	Type and mode: What is the effectiveness of various types/modes of delivery of behavioral therapies (e.g., group vs individualized, face to face vs remote)?
	Timing: When should behavioral therapy be introduced to effectively prevent or treat WR/IWL (e.g., preventive at weight plateau vs management after WR)?
Pharmacological	Type and dose: What is the effectiveness of various medication to manage WR/IWL (e.g., type of medication, single vs combination, effective dose)? Timing: What is the optimal time for medication/s to be introduced (e.g., preventive at weight plateau vs management after WR)?
Surgical	Revision type: What is the suitable type of revisional surgery for WR/IWL (e.g., better WL outcomes and lower complications)?

*WL* weight loss, *WR* weight regain, *IWL* insufficient weight loss, *WL* weight loss, *EWL%* excess weight loss percentage, *T2DM* type 2 diabetes, *HTN* hypertension, *OSA* obstructive sleep apnea, *GLP-1* glucagon-like protein-1, *PPY* peptide YY, *PA* physical activity, *QoL* quality of life, *pre-op* preoperative, *BS* bariatric surgery, *PA* physical activity

## Conclusion

BS remains effective and durable for treating obesity and ameliorating or resolving the obesity-related comorbidities. Understanding and tackling the WR/IWL after BS will likely require multi-pronged approaches. Standardized definitions could lead to more accurate and realistic estimates of the prevalence of these two conditions. The grouping together of WR and IWL as a common outcome or not depends on the context of the study; however, the current scoping review suggests that it would be better if WR and IWL were considered independently. Enhanced knowledge of the hormonal, psychological, behavioral, and surgical mechanisms contributing to WR/IWL could provide a more refined evidence base upon which appropriate prevention and management strategies could be premised. Better appreciation of the sequence and extents of the clinical impacts of WR/IWL will assist in determining the ideal timing and suitable intervention strategy/ies. Informed knowledge of the preoperative predictors of WR/IWL could aid to identify patients who are potentially at risk for both these conditions in order to offer them the necessary resources and counseling. Better understanding of the effectiveness and safety of the dietary, behavioral, pharmacological, and surgical prevention and management strategies will assist in the selection of intervention/s to mitigate WR/IWL. As the combination of such range of factors that contribute to WR/IWL are likely to be unique for each patient, applying such newly gained knowledge within a multidisciplinary team could be a step forward to individualized management plans tailored to patients in order to achieve resolution of WR/IWL.
